# Sprengel's Deformity Associated with Musculoskeletal Dysfunctions and Renal Anomalies: A Case Report

**DOI:** 10.1155/2012/398254

**Published:** 2012-02-28

**Authors:** Mohammad Hossein Kariminasab, Masoud Shayeste-Azar, Majid Sajjadi Saravi, Mehrdad Taghipour Gorgikolai

**Affiliations:** ^1^Department of Orthopedic Surgery, School of Medicine, Mazandaran University of Medical Sciences, Sari 4813894393, Iran; ^2^Cancer Research Center, Student Research Committee, Thalassemia Research Center, School of Medicine, Mazandaran University of Medical Sciences, Sari 4813894393, Iran

## Abstract

*Background*. Sprengel's deformity is a rare congenital anomaly of the shoulder girdle. The deformity is due to failure of descent of the scapula in intrauterine life. *Case Presentation*. We report a case of unilateral Sprengel's deformity associated with several other musculoskeletal and renal disorders consisting of absence of pectoralis major, weakness of trapezius and serratus anterior muscles, one kidney agenesis, and severe hydronephrosis of the other kidney in a 7-year-old boy. *Conclusion*. Sprengel's deformity can be associated with other musculoskeletal abnormalities and it is much more than a cosmetic problem.

## 1. Introduction

Sprengel's deformity is defined as abnormally high placed scapula [1–5]. Eulenberg described this condition in 1863 for the first time [2, 3]. It may involve one or both scapulas. The affected bone lies at a higher level than normal, and associated changes in axial rotation, shape, and size are usual [6]. The deformity results from failure to descent of the scapula from the C5-T1 position to the T2-T7 position at birth [2, 4]. The association of Sprengel's deformity with congenital scoliosis, fusion of the cervical vertebrae, and congenital heart disease has been described before [7]. But to our knowledge, its association with other musculoskeletal abnormalities or renal disorders has not been reported previously.

## 2. Case Presentation

**Figure 1:**
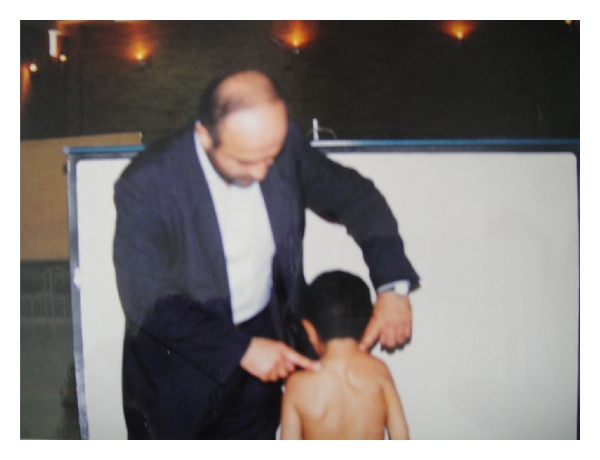
Photograph of the patient's body at 7 years of age. Congenital elevation of the right shoulder (Sprengel's deformity) apparent deformity is approximately severe and repair with surgery is needed.

**Figure 2:**
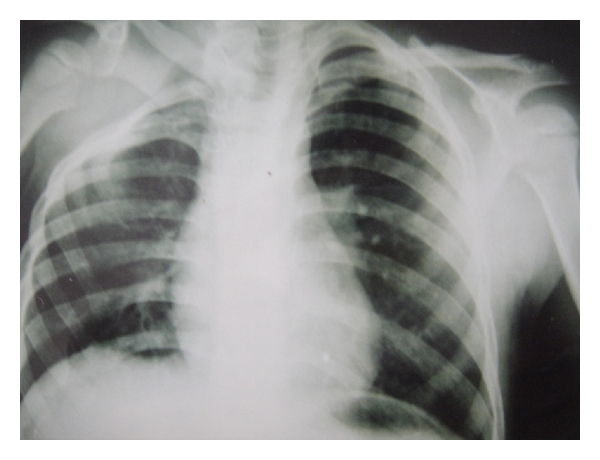
Anteroposterior radiography. This radiography shows right Sprengel's deformity associated with costal anomalies. Scapulovertebral bone is not seen.

A 7-year-old boy was brought by his mother to our outpatient clinic with the chief complaint of shortness of neck, head and neck deviation to the right side, and apparent limitations of right shoulder movements in abduction and elevation. His parents said that they had noticed the asymmetry in their child's shoulders when he was about 6 months, but they had been aware of his limited neck motion at much earlier time due to the difficulty in positioning the child for breast feeding. Familial history was unremarkable for any congenital disorder. On clinical examinations, right shoulder was apparently upper than the left shoulder in a standing position (Figure 1). His neck is shorter than normal in anterior view. Shortness of neck and elevation of right shoulder were apparent also from posterior view. Torticollis did not exist clearly, but there is a spastic muscle in the right side. Severe restrictions of active and passive rotation and lateral bending of the neck and abduction and external rotation of right shoulder were observed. He also had winging of the right shoulder that confirmed weakness of seratus anterior muscle. Ipsilateral pectoralis major was absent. Total muscular force of the right shoulder girdle is about 4+. Physical examination of the left shoulder is normal. The patient was admitted with the plan of surgery. Some routine diagnostic measures were done for him such as CXR, shoulders X-ray, and abdominal sonography. Radiological investigations (Figure 2) demonstrated an obvious skeletal deformity. Right shoulder was upper that the left side. Cervical rib was not seen in anterior view. A suspicious spina bifida in cervical vertebra existed. Right renal agenesis and severe hydronephrosis of the left kidney were diagnosed by abdominal ultrasonography. The patient went through the Wilkinson and Campbell scapulopexy operation. The shoulder range of motion increased significantly after surgery.

## 3. Discussion

The scope of abnormalities that were present in this case is different from what has been described by other authors. Congenital high scapula was an isolated problem in the cases introduced by S. V. Hodgson and McMurtry I. [8, 9]. Although Klippel Feil syndrome is usually responsible for the limitation of neck motion in these patients, we did not find any vertebral fusion in our patient and spastic neck muscles seemed to be responsible for this problem. Hegde and Shokeir [10] reported a case of Sprengel's deformity associated with pectoralis major agenesis. Most cases of absent pectoralis major muscle are associated with symbrachydactyly (Poland's disease), and renal failure is *also known to be accompanied* by this syndrome but the combination of congenital high scapula, congenital absence of pectoralis major and renal disorders has not been reported before.

## 4. Conclusion

An association of Sprengel's deformity with musculoskeletal dysfunction and renal anomalies is so rare and we could not find a similar case reported so far in orthopedic literature. Although this congenital abnormality is so rare but it should not be ignored in a society and we must find these cases and solve their problem either as functional or cosmetic aspect.
